# Applications of T_1_ and T_2_ relaxation time calculation in tissue differentiation and cancer diagnostics—a systematic literature review

**DOI:** 10.3389/fonc.2022.1010643

**Published:** 2022-11-24

**Authors:** Marta Micek, David Aebisher, Justyna Surówka, Dorota Bartusik-Aebisher, Michał Madera

**Affiliations:** ^1^ SoftSystem Sp. z o.o, Rzeszow, Poland; ^2^ Department of Photomedicine and Physical Chemistry, Medical College of The University of Rzeszow, Rzeszow, Poland; ^3^ Department of Biochemistry and General Chemistry, Medical College of The University of Rzeszow, Rzeszow, Poland

**Keywords:** MRI, cancer, diagnostics, T1 relaxation time, T2 relaxation time

## Abstract

**Introduction:**

The purpose of this review was to summarize current applications of non-contrast-enhanced quantitative magnetic resonance imaging (qMRI) in tissue differentiation, considering healthy tissues as well as comparisons of malignant and benign samples. The analysis concentrates mainly on the epithelium and epithelial breast tissue, especially breast cancer.

**Methods:**

A systematic review has been performed based on current recommendations by publishers and foundations. An exhaustive overview of currently used techniques and their potential in medical sciences was obtained by creating a search strategy and explicit inclusion and exclusion criteria.

**Results and Discussion:**

PubMed and Elsevier (Scopus & Science Direct) search was narrowed down to studies reporting T1 or T2 values of human tissues, resulting in 404 initial candidates, out of which roughly 20% were found relevant and fitting the review criteria. The nervous system, especially the brain, and connective tissue such as cartilage were the most frequently analyzed, while the breast remained one of the most uncommon subjects of studies. There was little agreement between published T1 or T2 values, and methodologies and experimental setups differed strongly. Few contemporary (after 2000) resources have been identified that were dedicated to studying the relaxation times of tissues and their diagnostic applications. Most publications concentrate on recommended diagnostic standards, for example, breast acquisition of T1- or T2-weighted images using gadolinium-based contrast agents. Not enough data is available yet to decide how repeatable or reliable analysis of relaxation times is in diagnostics, so it remains mainly a research topic. So far, qMRI might be recommended as a diagnostic help providing general insight into the nature of lesions (benign vs. malignant). However, additional means are generally necessary to differentiate between specific lesion types.

## 1 Introduction

**Figure 1:**
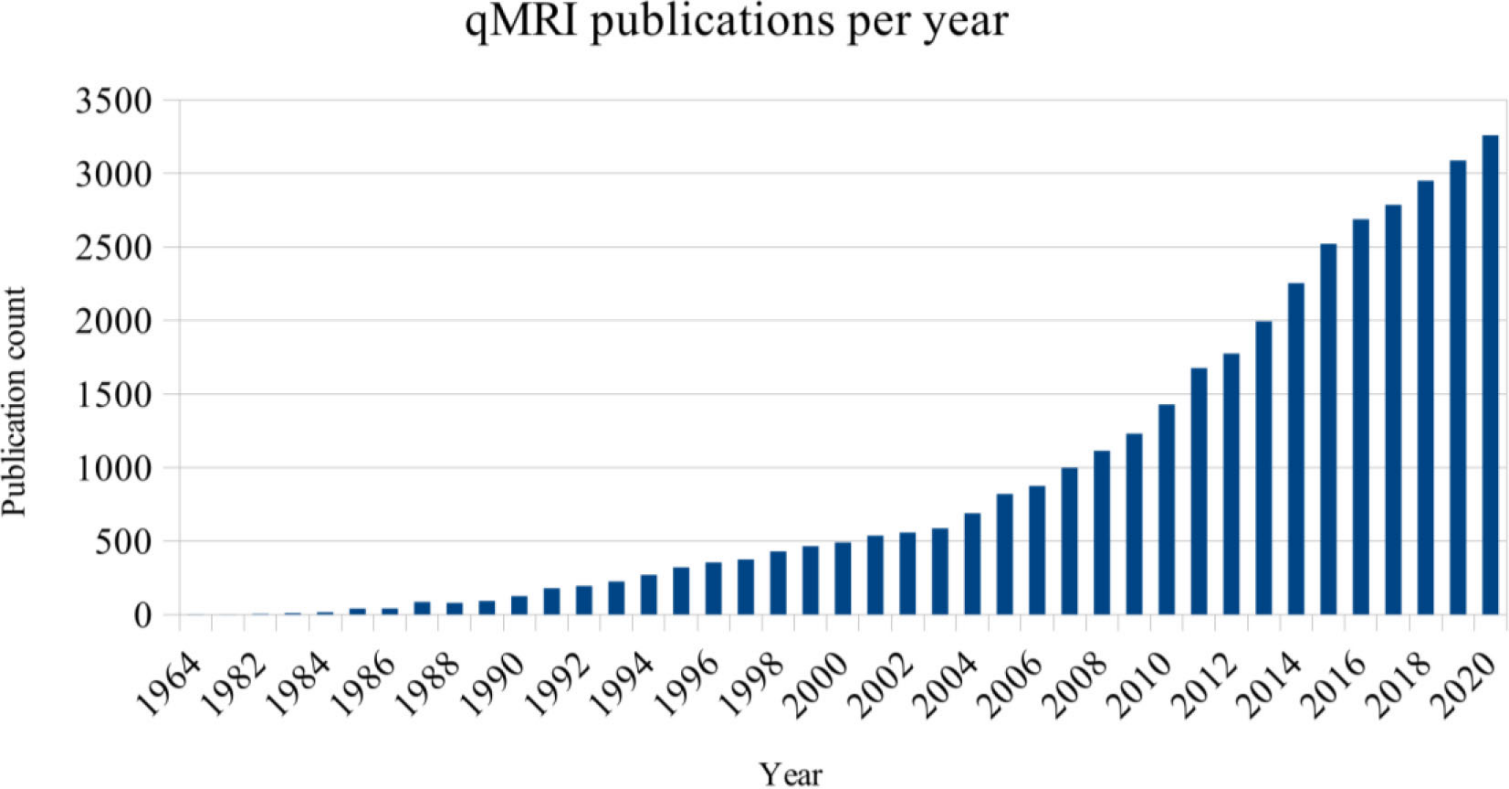
Number of entries in PubMed regarding “quantitative MRI”, by year of publication.

Magnetic resonance imaging (MRI) has been widely used since the seventies, and since almost the same time, quantitative magnetic resonance imaging (qMRI) techniques have been developed to assess the relaxatory parameters of tissues (**Figure 1**). Relaxation time calculation can nowadays be used in various applications, starting with relatively simple cases of cartilage degradation and ending up helping diagnose and contain the most dangerous cancers.

qMRI departs pretty significantly from the original approach, dedicated at best to obtain single T_1_- or T_2_-weighted images. In opposition to performing a single acquisition with set parameters of echo and repetition time, a series of scans are acquired in time with varying echo times for transverse relaxation or repetition times for longitudinal relaxation. Such an approach allows tracking the relative change of magnetization, beginning with the maximum (T_2_) or minimum (T_1_) signal strength at the beginning of a study and then calculating 33% (T_2_) or 67% (T1) of it, passing through consecutive time points. As a result, a signal change in time is acquired, and it is possible to calculate other parameters, such as the T_1_/T_2_ ratio or relaxation rates R_1_ and R_2_. Obtained results can be standardized, making it easy to compare relaxation curves and parameters between samples, devices, and studies.

As much as this description is simplified due to the multiplicity of sequences and practices in use, it should give an impression of one strength of qMRI: mathematics and models can, in certain cases, describe biological phenomena in more detail or maybe even completely different than a human eye. Due to that fact, the review has been written to get an overview of the potential usefulness of qMRI in clinical settings, as it might one day provide support for medical practitioners when it comes to tissue characterization and differentiation.

In this review, breast cancer and breast tissues were of primary interest. Due to limited literature on studies without the use of contrast agents, other tissues and organs were also considered; such an approach might be justified by the heterogeneous structure of the breast, built of epithelium, fat, and being very diverse when it comes to proportions of these components, which might affect results of relaxation time calculation. A few publications report successful differentiation or diagnosis of breast features when using qMRI, contrast-enhanced or not.

It must be stated that qMRI currently is not a recommended diagnostic method according to different authorities and organizations. The European Society of Breast Cancer Specialists (EUSOMA) 2008 produced a statement regarding, in their opinion, the best practices for diagnosing and treating breast cancer (1). While the publication mentions the acquisition of T_1_- and T_2_-weighted images using gadolinium-based contrast agents, all other practices are considered experimental and might be used, at best, as support for the basic analysis. Such techniques included diffusion-weighted imaging (DWI) and analysis of apparent diffusion coefficient (ADC), but there was no mention of qMRI, no matter the application.

Apart from these guidelines, the American Society of Breast Surgeons reminds us that MRI is not a modality of the first choice when screening or diagnosing patients unless other methods, such as x-ray mammography or ultrasonography, fail (2).

The European Commission Initiative on Breast Cancer (ECIBC) Guidelines Development Group (GDG) also presents a series of recommendations and suggestions regarding breast cancer screening, diagnosis, and treatment (3). Magnetic resonance imaging is generally described as a method “with very low certainty of evidence” compared with mammography (4).

It should be mentioned that qMRI is not the only modality currently being tested when it comes to screening for or diagnosing breast cancer. This is caused mainly by low mammography performance when applied to dense or extremely dense breast tissue. In such cases, many approaches have been tested, including ultrasound or X-ray-based techniques (5). Dynamic contrast-enhanced magnetic resonance imaging (DCE-MRI), currently a standard for high-sensitivity breast screening, comes with certain disadvantages, such as the need for intravenous contrast agent administration, which might result in rare, although possible, allergic reactions and is considered a more inconvenient protocol than mammography—a golden standard in screening programs.

Diffusion magnetic resonance imaging (dMRI) may be implemented in breast cancer imaging. The European Society of Breast Radiology (EUSOBI) recommends dMRI as a technique complementary to DCE-MRI. The organization opts for including standardized dMRI protocols in the Breast Imaging Reporting and Data System (BI-RADS). The main advantage of using dMRI together with other modalities is the acquisition of additional information on tissue metabolism and processes related to tissue perfusion, whereas the low resolution of dMRI makes it less informative if not used in conjunction with more precise imaging protocols.

Partridge et al. (6) described in detail the procedures and challenges related to dMRI. Fat suppression is essential but difficult due to its abundance in the breast. Despite that, and frequent other visual artifacts populating diffusion-weighted images, it still performs satisfyingly. One meta-analysis (7) reports pooled sensitivity of 84% when using dMRI to discriminate benign from malignant lesions. Another meta-analysis confirms good dMRI performance in supporting the analysis of DCE-MRI data (8). Multiple publications report correlations existing between dMRI-derived apparent diffusion coefficient (ADC) and tissue structure, where malignant changes, because of their cellular structure, result in lower ADC values (9, 10). Similar processes take part in non-malignant changes, such as ductal carcinoma *in situ* (DCIS), which can be differentiated from surrounding tissues with the use of dMRI (11).

The ADC has been suggested as a diagnostic biomarker in therapy assessment, allowing to differentiate between responders and non-responders to neoadjuvant treatment before changes in lesion size became visible (12). The ADC values obtained from samples were deemed repeatable, and their changes were significant after a month since the beginning of the therapy.

In one study, dedicated to performance assessment of mammography, DCE-MRI, and diffusion-weighted images, it was discovered that DCE-MRI was the most useful modality in cancer detection by professionals (13). The use of mammography resulted in the least precise predictions by observers, whereas decisions based on mammography together with diffusion-weighted and T2-weighted images were moderately correct. It means that if no DCE-MRI examination is possible, other techniques might still increase the sensitivity of screening.

Another review, by Amornsiripanitch et al. (14), talks specifically about non-enhanced MRI (without the use of contrast agents). Due to restricted water particle movement in cancerous tissues, malignant changes appear hyperintense in dMRI. The use of diffusion-weighted imaging, requiring less time, effort, and financial resources than DCE-MRI, seems to be a promising supporting technique for mammography screening with 89% effectiveness of contrast-enhanced methods (15). It also seemed that dMRI was resistant to factors affecting other modalities and consisting of menopausal status, menstrual cycle, breast density, or lesion size, although the last one might still be important due to the low resolution of dMRI (16, 17).

Other approaches to breast imaging involve coupling MRI together with positron emission tomography (PET) or performing multiparametric MRI (mpMRI), based on a simultaneous analysis of images from complementary modalities (18).

The use of complementary imaging methods is also possible in the screening and diagnosis of prostate and brain cancers and tumors (19, 20). Specific methods have not yet gained similar popularity but seem to be employed in specific clinical trial settings (21). These methods include dynamic susceptibility contrast MRI (DSC-MRI), chemical exchange saturation transfer (CEST), and hyperpolarized MRI.

The scarcity of qMRI publications regarding breast tissues may be a result of factors such as accessibility to equipment and software capable of making high-quality measurements. Performing non-standard procedures requires highly trained staff, and any studies involving patients by engaging them in full or by using samples obtained from biopsies requires additional approvals from relevant ethics committees, not to mention the patients’ consent.

By performing this review, we tried to get an overview of possible applications and capabilities of qMRI as well as verify how easy or difficult it is to query scientific databases and how much relevant information can be acquired. By carefully preparing search phrases and specification of acceptable search criteria, such as tissue and modality type, multiple unrelated publications were filtered out not because of their improper quality but often due to unfortunate wording or unspecific writing. Through a systematic review, it is also possible to learn predominant practices when performing qMRI experiments, which might be a suggestion for future researchers, as there are numerous possible combinations of qMRI scan parameters and procedures.

The systematic literature review methodology used in this work is presented in Section 2. Outlined was the justification for the literature review with the research questions and search query statements. The overview of the search process was summarized with the PRISMA diagram shown in Section 3. Next, Section 4 shows previous work related to reviews on the main subject topic, followed by the analysis of the original publications. The conclusions are presented in Section 5, which provides information on good practice and research trends in applications of calculating the relaxation times T1 and T2 in tissue differentiation and cancer diagnosis.

## 2 Structure of the review

An attempt was made to follow recommendations regarding conducting systematic reviews, although not all were met due to limited time and resources. As guidance, the Cochrane Handbook (22) served as a valuable source of knowledge and suggestions from BioMed Central (23).

### 2.1 Justification for the review

Application of qMRI might result in numerous benefits when properly applied, such as

- ease of data standardization and comparison due to calculation of relative signal intensity and T_1_/T_2_ proportions,
- support in ROI selection based on tissue properties, especially in areas where it is difficult to tell apart tissue types or malignant changes on weighted and other MRI scans,
- no need for contrasting agents when assessing patients with allergy to gadolinium or kidney issues; go-to solution for *ex vivo* studies, where DCE-MRI (dynamic contrast-enhanced MRI) cannot be applied.

All the promises and benefits make it necessary to ask questions such as the following: is qMRI really that efficient when differentiating tissues, or could it cause more harm than good when applied, especially incorrectly and without sufficient knowledge? Are T_1_ and T_2_ constants uniform or diverse enough across tissues, samples, and patients to be indicators of malignancies?

### 2.2 Questions asked

Before the beginning of the review process, the following questions were formulated:

1. What approaches are in use when applying qMRI to tissue differentiation?
2. Which tissues and organs are the most frequently analyzed?
3. Does practice or efficiency differ for epithelial tissue compared with other tissue types?
4. Are T1 and T2 time calculation results significant and comparable between samples, patients, and studies?
5. Which database or search engine is the complete source of topic-related literature?

The reasoning behind categorizing by tissue was forced due to the diverse structure of the human body. Depending on the tissue, different approaches might be used to compensate for specific phenomena, such as blood flow in the myocardium or oxygen and carbon dioxide exchange in the lungs.

A more detailed description of subjects of interest is found in **Table 1**, where the research questions were formulated to fit the Population-Intervention-Comparison-Outcome-Context (PICOC) scheme.

**Table 1:** Review questions in light of the PICOC scheme.

Population	Patients and study participants of all genders and ages; any human tissue samples being analyzed—healthy and bearing signs of pathological processes. Exceptionally, human cell cultures.
Intervention	Calculation of T_1_ and T_2_ constants and/or T_1_/T_2_ ratio based on qMRI data to identify or differentiate tissues.
Comparison	Between different tissues or in a single tissue before and after treatment. Intra- or inter-patient.
Outcomes	A positive outcome of a study would be a way to efficiently differentiate between samples using qMRI techniques and individually formed criteria.
Context	Quantitative experiments take place mostly in clinical settings, which makes it difficult to obtain a large number of participants and later access acquired data due to their sensitivity. As a result, there is a risk of sample groups being limited or too small to be significant. Relaxation time analysis is also not a diagnostic standard, so it is most often a subject of academic studies, not clinical studies and trials.

### 2.3 Source selection

Based on personal experience and suggestions from specialists, the largest biomedical databases were chosen for the review:

- PubMed (24)
- Elsevier [Scopus (25)/Science Direct (26)]

### 2.4 Search strategy

The search was performed in English, using queries formed and refined to retrieve as many relevant studies as possible. Although it would be possible to include other languages, they might not necessarily be known by readers, and thus it would not be easy to follow and validate such references.

Sensitivity was more critical than specificity, so there was a higher tendency to include irrelevant sources in initial lists than unintentionally discard relevant papers.

The following queries were used in all databases:

1. “(t1 relaxation OR t2 relaxation) AND (malignant OR benign)”
2. “(t1/t2 ratio OR t1 relaxation or t2 relaxation) AND differentiation”
3. “(t1/t2 ratio OR t1 relaxation or t2 relaxation) AND differentiation AND (benign OR malignant)”
4. “t1/t2 AND relaxation AND breast”
5. “breast AND (t1 relaxation OR t2 relaxation) AND differentiation
6. “breast AND qMRI”
7. “breast AND (quantitative MRI)”
8. “magnetic resonance imaging AND breast AND quantitative”

The search was narrowed to sources published in or after the year 2000. Sporadically earlier publications or articles not meeting all of the criteria might be mentioned in the literature review part and results due to their overall value as reviews or novelties, but such cases are clearly stated.

Matches were valid only when found in titles, abstracts, and keywords. Additionally, references used in relevant publications were manually analyzed in search of further related sources, and their relatedness was again assessed based on title, abstract, and, if necessary, full-text analysis, as their numbers were lower while it was more likely that some of them would be relevant.

Queries based on elimination, using “not” and terms referring to modalities and technologies, were discarded due to the unverifiable elimination of seemingly too many results.

The final collection of publications was assembled after performing all queries and eliminating duplicate entries.

### 2.5 Inclusion and exclusion criteria

Inclusion and exclusion criteria were defined to extract a set of similar qMRI techniques with diagnostic potential.

It was expected that every primary publication provided sufficient information about the hardware and software used (scanner brand and basic parameters like operating magnetic field and type of coil used, image acquisition, and data processing software) as well as applied signal sequences.

T_1_ and T_2_ constants should be calculated based on a series of qMRI scans performed over time without using contrasting agents, at least in part of a study. Using gadolinium, iron or any other substances would make a potential comparison of study results difficult, as contrasting agents work by altering the relaxation times of tissues. In that case, T_1_ and T_2_ values and their ratio would be different than during normal measurements.

There was no requirement to report exact formulas used to calculate T_1_ and T_2_ for as long as possible to trace down software used for calculations and methodology applied if there was any freedom to use software modules. This did not apply to computing environments and programs where users needed to provide their code. In such cases, the formula or model should have been referenced optimally with a fitting algorithm and other operations affecting the data.

Approaches targeted at healthy tissue differentiation were included, as well as differentiation between healthy and benign or malignant changes. Statistical analysis of researched differences was not required but considered a disadvantage if missing. Comparisons of T_1_/T_2_ acquisition methods with other diagnostic techniques were also accepted if all other criteria were met but were not analyzed due to not being the main topic of the review.

No meta-analysis was attempted, and the purpose of the review was not to grade existing works in any way. Efficiency and appropriateness of described techniques were noted in the form of comments in tables based on the precision of reporting, size of the study group, and results of any statistical analyses if present. Diagnostic aspects of studies were also noted whenever available. Some of them, especially human-dependent factors, might significantly affect obtained results. **Table 2** contains information regarding the reasoning behind operations such as ROI selection, which can be directed purely by human judgment or computer-assisted.

**Table 2:** Methodology and approaches to analysis in the detailed review studies.

⁠⁠Publication	ROI selection	Conclusions
Breast		
Relaxation times of breast tissue at 1.5T and 3T measured using IDEAL (27)	Menstrual cycle considered: patients scanned no more than 2 days apart each; ROI: average of three points in fat or glandular area. Size varied—drawn to maximize the area of homogeneous tissue.	An increase in a magnetic field leads to relaxation time decrease; IDEAL in the same field increased relaxation times. because it removes the water signal from adipose tissue (leaving only signal from actual fat). Significant differences between fields and tissues ONLY for T1, not significant for T2.
Longitudinal and Multi-Echo Transverse Relaxation Times of Normal Breast Tissue at 3 Tesla (28)	Single 3-mm-thick coronal slice midway between nipple and chest wall. Voxel-wise relaxation maps.	Knowledge of fat and water T1 times allows efficient fat and water signal suppression. The resulting values were 15%-30% higher than in a similar study using 3T (18)⁠, but the earlier one used only two-time points for T2. Very few participants (5-6). Better contrast against fat visible for very dense fibroglandular tissue.
T1 and T2 temperature dependence of female human breast adipose tissue at 1.5T: groundwork for monitoring thermal therapies in the breast (29)	Contained 30 voxels (2 × 2 × 10 mm)?, manual placement in adipose regions	Dependence of T1 and T2 on temperature showed little inter-sample variation
Changes of T2 relaxation time from neoadjuvant chemotherapy in breast cancer lesions (30)	Consensus between two radiologists; drawn on T2 images if lesions were visible or on a fusion of T2 and DWI images. Avoiding necrotic and cystic areas. Mean value from three different regions of a lesion.	Significant differences before and after; also significant between responders and non-responders after, but not before
Role of quantitative analysis of T2 relaxation time in differentiating benign from malignant breast lesions (31)	1-9 cm^2^, depending on tumor size, based on STIR	Large overlap between T2 range for benign and malignant samples. Significant differences between malignant and benign samples, but not between different types of malignancies; shorter T2 for malignant. Large overlap between groups makes clustering inefficient.
Lung		
T2 mapping of CT remodeling patterns in interstitial lung disease (32)	“as large as possible, not less as 100 mm2, and positioned to avoid major heterogeneities: large blood vessels, main airways, or motion mismatch that could not be corrected”, with reference to CT scans	Significant differences in T2 times depending on the amount of fibrous tissue; differentiation between healthy and pathological tissue.Significant differences between normal and pathological tissues; between values of parenchymal features (GGO, RE, HC);also, between left and right lung for GGO and RE
Prostate		
Measurements of T1-relaxation in *ex-vivo* prostate tissue at 132µT (33)	Classified into normal and cancerous based on expert’s observation. Mix of qMRI and NMR to obtain better SNR	T1 contrast is increased at very low field (below 1mT), but low SNR is an issue. Large T1 variability between patients, but it should be sufficient to have enough intrapatient contrast to tell cancer apart from normal tissue. Shorter T1 for cancer.
Relationship between T2 relaxation and apparent diffusion coefficient in malignant and non-malignant prostate regions and the effect of peripheral zone fractional volume⁠ (34)	Freehand ROI around dominant tumor nodule, with reference to ADC maps	Significant differences in T2 for prostate zones and tumorSignificant differences between tissue areas around tumors, and additionally correlation between T2 values and diffusion coefficients.
Changes in apparent diffusion coefficient and T2 relaxation during radiotherapy for prostate cancer (35)	By a radiologist/based on the decreased intensity of T2 signal, DCE, and consistency with the previous biopsy	In some cases, therapy resulted in significant changes in T2
Rotating frame relaxation imaging of prostate cancer: Repeatability, cancer detection, and Gleason score prediction (36)	“drawn on TRAFF, T1rcw,and T2 images using anatomical T2wi and prostatectomysections as the reference”	T2 can be used as a parameter to discriminate between healthy and malignant prostate tissues.
Skin		
*In vivo* morphological characterization of skin by MRI micro-imaging methods (37)	“linear regions of interest of 40 × 1 pixels parallel to the surface (6 mm × 50 µm) located in the center of the area imaged to a depth of 2.5mm”	qMRI, especially at high resolution, allows for efficient skin assessment.Mean values for skin layers are provided, but no statistical analysis was performed.
Kidney		
Quantitative versus qualitative methods in evaluation of T2 signal intensity to improve accuracy in diagnosis of pheochromocytoma (38)	Images evaluated by two radiologists; lesions classified as homo-or heterogeneous. ROIs were approaching, but not including borders of lesions.	Lesion intensity was analyzed as relative to CSF and other organs, with no values assumed as arbitrary. An unusual approach among other studies, but allowed to achieve good classification results (adrenal to muscle (81%) and adrenal to liver best) when distinguishing from other adrenal lesions. Alternatively, in some cases, lesion discovery failed at all, whereas they were visible in qualitative analysis.
Liver		
Characterization of focal liver lesions using quantitative techniques: comparison of apparent diffusion coefficient values and T2 relaxation times (39)	ROI included the largest possible part of a lesion, avoiding blood vessels, necrosis, artifacts, and partial volume effects. ROC to define ADC and T2 cutoff between benign and malignant lesions.	T2 had much better AUC than ADC for differentiating between benign and malignant lesions.Mean T2 was lower for malignant than benign lesions.AUC for diagnosing malignancy was 0.932 with sensitivity of 99% and specificity of 80.8%. No possibility of differentiating between specific lesion types due to large overlap.
Differentiation of Hepatocellular Carcinoma and Hepatic Metastasis From Cysts and Hemangiomas With Calculated T2 Relaxation Times and the T1/ T2 Relaxation Times Ratio (40)	Manual placement of the largest rectangular ROI within a lesion, by a single investigator. If regions of increased T2 (cysts/necrosis) were present, the ROI was placed along the border, without the center.	The T1/T2 ratio allows differentiating between cysts, hemangiomas, and solid lesions. No overlap between ratios for benign and malignant lesions. Almost 100% sensitivity and specificity for classification based on a ratio. Only lesions larger than 1cm in the largest dimension.
Discrimination of benign from malignant hepatic lesions based on their T2-relaxation times calculated from moderately T2-weighted turbo SE sequence (41)	Two measurements for each lesion. Largest possible ROI, excluding cysts and necrotic regions. Also, reference ROIs from normal liver tissue.	Differentiation with the threshold between 67 and 116 ms resulted in a classification of benign and malignant lesions with a sensitivity of 90% and specificity of 94%. Statistically significant differences between malignant and benign lesions. Despite this fact, the authors still recommend using qMRI as support for other techniques, such as DCE-MRI due to T2 range overlap.
Hepatic malignant tumor vs. cavernous hemangioma: differentiation on multiple breath-hold turbo spin echo MRI sequences with different T2-weighting and T2-relaxation time measurements on a single slice multi-echo sequence (42)	Largest lesion of a patient selected for assessment. Section with the largest tumor dimension. An elliptical or circular region with maximal inclusion of tumor but excluding partial volume averaging with the surrounding liver.	Not only T2 values were compared but also changes in signal intensity along the entire timeline, and intensity at certain echo times showed better clustering potential than others. Fat suppression provided better results than when not applied.
Recurrent hepatocellular carcinoma versus radiation-induced hepatic injury: differential diagnosis with MR imaging (43)	ROIs included parenchyma, irradiated areas, and HCCs.	Patterns of hypo- and hyper-intensity are different for studies with and without contrast. To make HCCs stand out in the irradiated area (after radiation therapy), the use of contrast was beneficial (no significant differences in intensity without it).
Differentiation of focal hepatic lesions in MR imaging with the use of combined quantitative and qualitative analysis (44)	For each of two echoes two measurements were taken and averaged. ROI covering as much tumor as possible, limited to solid parts of tissue	Qualitative analysis was used to differentiate between solid tumor types by the agreement of two radiologists.Significant differences between solid tumors and other lesion types – optimal threshold of 116 ms (96% sensitivity, 93% spec.)

## 3 Search strategy summary

Identification of topic-related publications has proven more complicated than was initially assumed. Although many original articles were identified based on a defined search strategy utilizing titles, abstracts, and keywords, further analysis showed that roughly 20% of initially included articles were relevant to the review’s topic. That finding aligns somewhat with the review strategy, putting more significance on sensitivity than specificity.

The most frequent feature leading to the elimination of an article was unspecific vocabulary. Authors frequently used T_1_ and T_2_ relaxation time-related terms, but their works described only the acquisition of T_1_- or T_2_-weighted images; another case was low precision of titles and abstracts when it came to describing modalities—multiple original publications were discarded due to using nuclear magnetic resonance (NMR) or magnetic resonance spectroscopy (MRS) instead of qMRI.

The review was primarily targeted toward breast and epithelial tissue, so specific searches were done to investigate these matters in detail. It seems possible that specific searches toward other organs and tissues would result in an even more extensive collection of original works of interest. Also, many additional results were retrieved when using the last query (“quantitative magnetic resonance imaging”) instead of the abbreviation (qMRI). It could suggest that using the full names of technologies in queries might be a good practice. Unfortunately, in this case, it resulted in 228 positions either not being related to relaxometry at all (interpreting only “magnetic resonance imaging” probably without a match for “quantitative”) or involving the use of contrast agents (about 42% of 228). The rest of the publications did not meet the other requirements, leaving only two papers seemingly relevant: one about breast cancer-related lymphedema (BCRL) and the other one (discarded) based on human cell culture and rat xenografts.

**Figure 2:**
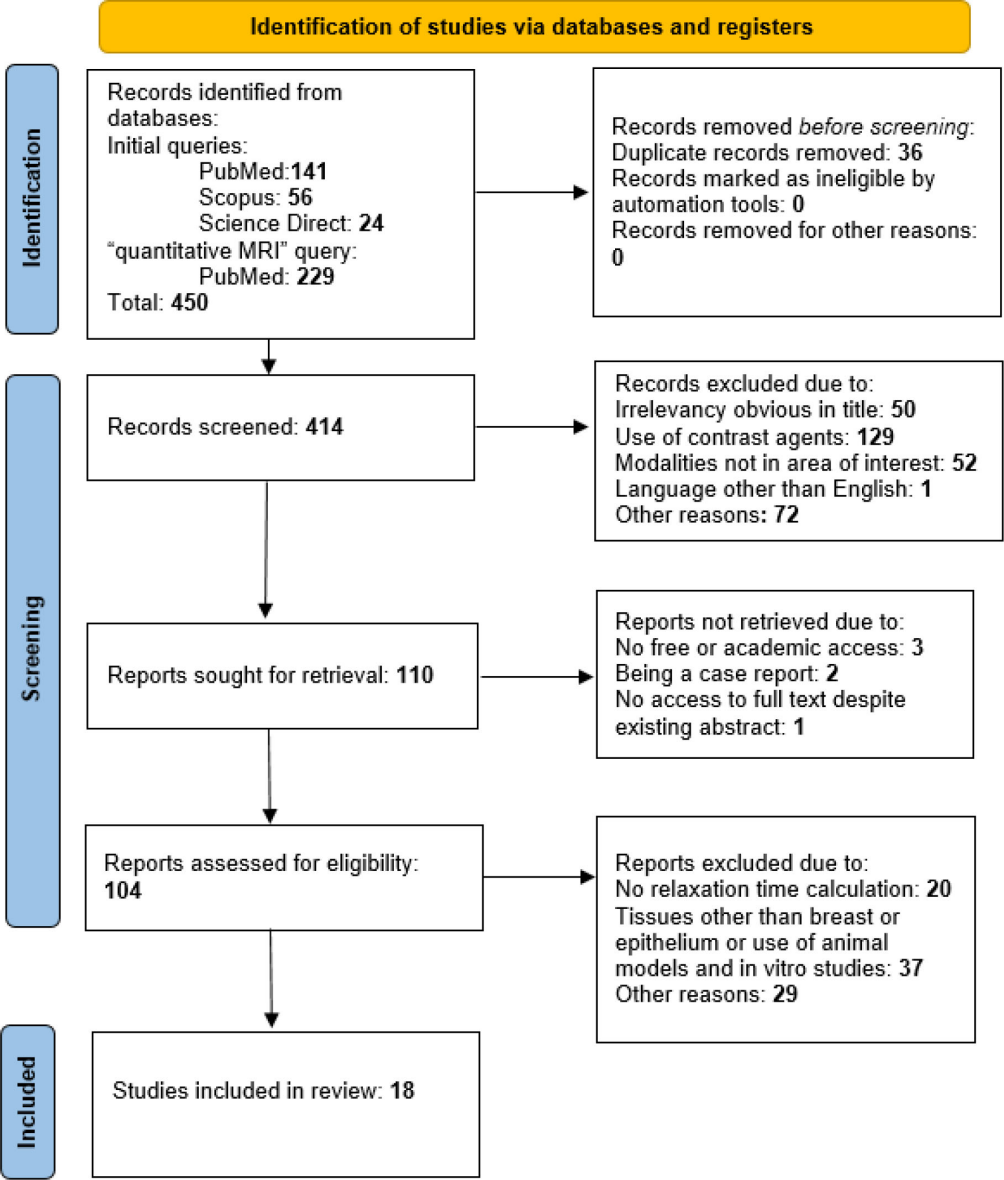
The review process is summarized on a PRISMA diagram.

The Preferred Reporting Items for Systematic reviews and Meta-Analyses (PRISMA) diagram (45) was included to provide an overview of the search process and is presented in **Figure 2**. The diagram shows results obtained after using the initial set of queries listed in point 2.4 and results from the query “quantitative magnetic resonance,” which was added later. No automation tools were used to perform the identification or screening of retrieved publications.

## 4 Literature review

### 4.1 Reviews and systematic reviews so far

Five reviews that meet the search criteria have been identified (46–50). The first one summarizes current techniques of T_1_ and T_2_ time calculation at a magnetic field of 3T. It also reports T_1_ and T_2_ values for different tissue types—white/gray matter, CSF, muscle, myocardium, fat, and others. The authors describe multiple aspects to consider when calculating relaxation constants, such as signal noise, partial volume effects, or magnetic field inhomogeneity as sources of large differences between reported T_1_ and T_2_ values for different tissues.

Another review has been mentioned as a reference in (46), related to methods of calculation of T_1_ (51). It is not related to tissue classification and is dated outside the chosen time range (2000–now), but it provides a complex overview of the literature describing approaches up until 1999. It might be of interest to those investigating differences between T_1_ times acquired with different techniques.

Wolf et al. (47) presented a review of relaxation time analysis in kidneys, reporting multiple studies and their results regarding both longitudinal and transverse relaxation. Basic qMRI principles and sequences used for renal imaging are mentioned. Multiple factors affect possible results, such as fasting, hydration or oxygenation level, modulating diuresis speed, and fluid retention in necrotic tissue. Such factors are highly specific and do not apply to the imaging of most other organs. In general, the authors conclude the potential usefulness of renal qMRI, although it is strongly susceptible to physiological and pathological alterations, which should always be accounted for.

For cartilage degradation studies, a review has been published (50) that investigates the impact of preexamination activity and exercise on the results of T2 measurement. The review shows that activity might cause water particle movement in cartilage, which affects its relaxatory parameters. Thus, pre-scan procedures should be implemented to reduce patient movement, as it could be a source of variability in T2 values obtained by different teams.

The last review, published in two parts, provides complex information about current cardiac imaging standards concerning T1 estimation. The authors summarize methodological aspects of myocardial T1 and ECV, such as sequence choice (with MOLLI or SASHA being the most popular ones) or motion correction. It is emphasized in the paper that precise comparisons between studies are possible when the same protocols are being used to obtain results. Due to that fact, T1 analysis has a significant supportive value in cardiac studies, but it cannot be used as a standalone diagnostic parameter.

### 4.2 Original publications

**Figure 3:**
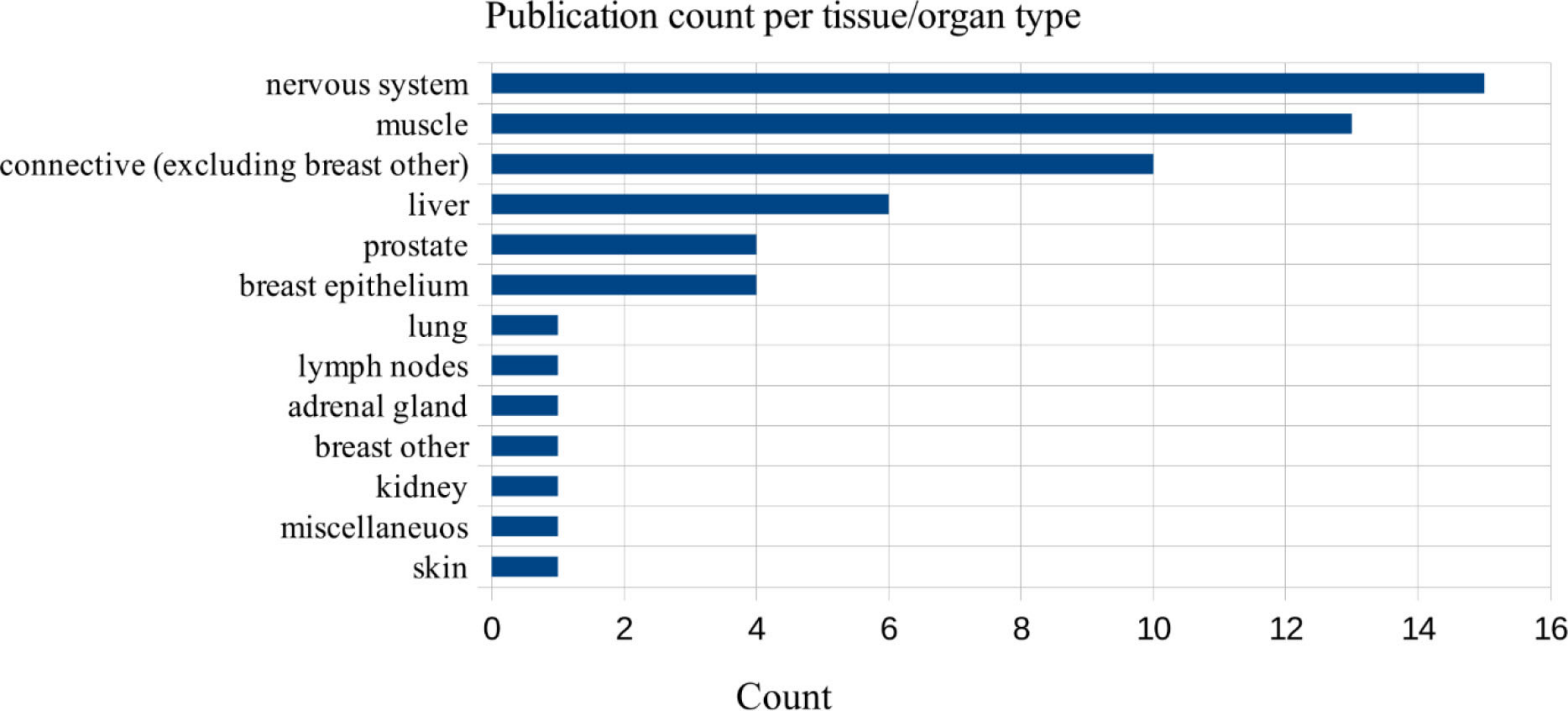
The number of studies in a detailed review, grouped by organs and tissues, total = 59.

The number of relevant sources retrieved was not high but sufficient to draw basic conclusions on the use of relaxation times in diagnostics and tissue differentiation. Based on the material, it can be seen that relaxation analysis is practiced more often on some tissues than others. Most of the 59 publications (25%) were dedicated to the nervous system, particularly the brain. qMRI was also frequently used when studying connective tissues, such as cartilage (17%) or muscle tissue, including myocardium (22%). Another 10% of studies treated the liver. Studies dedicated to quantification of other tissues and organs (breasts, prostate, kidneys, and others) were encountered sporadically when using queries 1–3 (see *Search strategy*), as shown in **Figure 2**. Queries formulated specifically to retrieve breast-related studies allowed us to identify a higher number of valuable articles (**Figure 3**).

The structure and data provided by authors of publications were strongly diverse, which suggests no “golden rule” for conducting this kind of study. **Tables 3**, **4** summarize experimental setups from articles chosen for the detailed review.

**Table 3:** Technical aspects of publications chosen for the detailed review.

Publication	Year	Field	Scanner	Sequences			Scanner or data analysis software
				T_1_	T_2_	Other	
Breast							
Relaxation times of breast tissue at 1.5T and 3T measured using IDEAL (27)	2006	1.53	Echospeed whole-body magnet, GE Healthcare, Waukesha, WI, USA)	FSE-IR	Two Hahn echo scans	IDEAL	Matlab (The MathWorks, Natick, MA)
Longitudinal and Multi-Echo Transverse Relaxation Times of Normal Breast Tissue at 3 Tesla (28)	2010	3	Philips Intera 3T (Philips Healthcare, Best, the Netherlands)	Inversion recovery-prepared multi-shot spin-echo EPI	Spin echo		Matlab (The MathWorks, Natick, MA)
T1 and T2 temperature dependence of female human breast adipose tissue at 1.5T: groundwork for monitoring thermal therapies in the breast (29)	2015	1.5	Achieva (Philips Healthcare, Best, the Netherlands)	Two-dimensional inversion recovery TSE scans with SPIR-WS	Multi-spin echo	T_2TSE_	
Changes of T2 relaxation time from neoadjuvant chemotherapy in breast cancer lesions (30)	2016	1.5	GE Signa, Milwaukee, WI, USA	FSE, STIR	FSE	T1W, T2W; 3D after contrast	SPSS software, ver. 11.5 (SPSS Inc., Chicago, Il, USA), Functool T2 mapping software
Role of quantitative analysis of T2 relaxation time in differentiating benign from malignant breast lesions (31)	2018	1.5	SIGNA™ Infinity; GE Medical Systems	Axial FSE T1-weighted	Sagittal fat-suppressed T2-WI	Axial DWI, axial short-time inversion recovery (STIR)	Functool (Advantage Workstation 4.3 (GE Medical Systems));SPSS Inc., Chicago, IL, USA
Lung							
T2 mapping of CT remodeling patterns in interstitial lung disease (32)	2015	1.5	Magnetom Aera, Siemens Medical Systems		Multi-echo single-shot turbo spin echo sequence	CT	MRmap for IDL8.3 Software,R 2.15.1 (R Foundation for Statistical Computing, Vienna)
Prostate							
Measurements of T1-relaxation in *ex-vivo* prostate tissue at 132µT (33)	2012	132µT	Techmag Orion system	SQUID			No data/exponential fit for T1
Relationship between T2 relaxation and apparent diffusion coefficient in malignant and non-malignant prostate regions and the effect of peripheral zone fractional volume (34)	2013	3	Achieva (Philips Medical Systems, Best, Netherlands)		FSE	T1W, T2W	SPSS^®^ v. 19 for Windows (IBM Corporation, Portsmouth, UK)
Changes in apparent diffusion coefficient and T2 relaxation during radiotherapy for prostate cancer (35)	2013	1.5	Signa, General Electric Medical Systems, Waukesha, WI		Magnetization‐prepared spiral imaging pulse sequence	2‐weighted fast‐spin‐echo (FSE), DWI	MIPAV (National Institutes of Health, Bethesda, MD), Origin software (OriginLab, Northampton, MA)
Rotating frame relaxation imaging of prostate cancer: Repeatability, cancer detection, and Gleason score prediction (36)	2016	3	Ingenuity PET/MR, Philips, Cleveland, OH		GraSE	T2W, T1rho	GraphPad Prism, version 5.00 (GraphPad Software, San Diego, CA), MATLAB (MathWorks Inc., Natick, MA)
Skin							
*In vivo* morphological characterization of skin by MRI micro-imaging methods (37)	2004	2	Magnex Scientific Ltd., Oxford, England	GE		MT, T1W, T2W	Customized imaging console (SMIS Ltd.)
Kidney							
Quantitative versus qualitative methods in evaluation of T2 signal intensity to improve accuracy in diagnosis of pheochromocytoma (38)	2015	1.5	Signa, GE Healthcare		Breath-hold SSFSE or respiratory-triggered frequency-selective fat-suppressed fast recovery FSE	RARE T2W, chemical shift imaging	SPSS (SPSS Inc., Chicago, IL, USA), Matlab (version 2014b, MathWorks).
Liver							
Characterization of focal liver lesions using quantitative techniques: comparison of apparent diffusion coefficient values and T2 relaxation times (39)	2012	1.5	Magnetom Avanto, Siemens Medical Solutions, Erlangen, Germany)		Breath-hold dual echo T2W TSE (with AND without contrast)	DW-SS-EPI, 3D DCE-MRI	ADC: Leonardo Workstation, Siemens Medical Solutions, Erlangen, Germany), Statistica 10.0, Microsoft Excel
Differentiation of Hepatocellular Carcinoma and Hepatic Metastasis From Cysts and Hemangiomas With Calculated T2 Relaxation Times and the T1/ T2 Relaxation Times Ratio (40)	2006	1.5	Philips Intera, Philips Medical Systems of North America, Andover, MA, USA)	Mixed-TSE	Mixed-TSE		DICOM processing on PC using MathCAD 2001i (MathSoft, Cambridge, MA, USA), SAS 8.02 (SAS Institute, Cary, NC, USA)
Discrimination of benign from malignant hepatic lesions based on their T2-relaxation times calculated from moderately T2-weighted turbo SE sequence (41)	2002	1.5	Gyroscan ACS NT, Philips, Eindhoven, The Netherlands		Double echo TSE		
Hepatic malignant tumor vs. cavernous haemangioma: differentiation on multiple breath-hold turbo spin echo MRI sequences with different T2-weighting and T2-relaxation time measurements on a single slice multi-echo sequence (42)	2002	1.5	Gyroscan ACS NT, Philips Medical Systems, Best, The Netherlands		8-echo TSE	With or without fat suppression	
Recurrent hepatocellular carcinoma versus radiation-induced hepatic injury: differential diagnosis with MR imaging (43)	2001	1.5	Signa Advantage (GE Medical Systems, Milwaukee, WI, USA)	Spin-echo	Spin-echo	DCE-MRI, T1W, T2W	T2 fitting with scanner built-in software
Differentiation of focal hepatic lesions in MR imaging with the use of combined quantitative and qualitative analysis (44)	2007	1.5	Gyroscan ACS NT, Philips	T1 FSMPGRE	TSE, dual-echo TSE, T2 STIR	Dynamic FSMPGRE	Microsoft Excel, Statistica 6.0 (StatSoft Poland)

**Table 4:** Biomedical aspects of publications chosen for the detailed review.

Publication	Tissue/organ	Subject	Environment	Reported T2 values [ms]				Reported T1 values [ms]	Participants: number/age [years]
Breast									
(27)	Breast adipose and fibroglandular tissue	Normal tissue characterization	*In vivo*	Fat	1.5T	IDEAL		296.01 ± 12.94	5/36.4 ± 12.6
						Non-IDEAL	53.33	372.04 ± 8.6	
					3T	IDEAL		366.78 ± 7.75	
						Non-IDEAL	52.96	449.27 ± 26.09	
				Glandular	1.5T	IDEAL		1,266.18 ± 81.8	
						Non-IDEAL	57.51	1,135.98 ± 151.37	
					3T	IDEAL		1,444.8 ± 92.7	
						Non-IDEAL	54.36	1,324.42 ± 167.63	
(28)	Adipose and fibroglandular tissue	Normal tissue characterization	*In vivo*	154 ± 9 adipose,71 ± 6 fibroglandular				423 ± 12 adipose,1,680 ± 180 fibroglandular	6/34 ± 6, 38-49
(29)	Connective (adipose) tissue in breast	Dependence of adipose tissue T2 on temperature	*ex vivo*	55 to 100 for temp. 25°C–65°C				200–550 ms for temp. 25-65	21-56, mean 30
(30)	Breast	Lesion response to neoadjuvant therapy measured with T2 constant	*In vivo*	81.34 ± 13.68 ms pretreatment, 64.50 ± 8.71 ms posttreatment					34-70, mean 55.2
(31)	Breast	benign and malignant changes in breasts, mostly invasive ductal carcinomas	*In vivo*	Benign:fibrodenomas: 92.53 ± 22.76papillomas:84.36 ± 14.69fibrocystic adenosis:103.56 ± 4.17Malignant:inv. ductal carc.: 80.64 ± 10.16inv. Lobular carc.: 76.87 ± 14.01ductal carc. *in situ*: 82.29 ± 12.51					67/mean 50.7 ± 17.3,(26-74)
Lung									
(32)	Epithelium—lung	Differentiation and characterization of lung tissues in pneumonia	*In vivo*	Median 41, 38-49					Six men and six women, with a mean age of 62(47-81)
Prostate									
(33)	Prostate	Contrast between normal and cancerous prostate tissue	*Ex vivo*					41–86, according to authors shorter for cancer, but randomly checked samples often show opposite results	No data, 35 *ex vivo* specimens
(34)	Prostate	Malignant vs. non-malignant prostate regions	*In vivo*	PZ: 149 ± 49 ms, TZ: 125 ± 26 ms, tumor: 97 ± 23 ms PZ = peripheral zone, TZ = transition zone					48–83 years (mean age 67 ± 8 years)
(35)	Prostate	Prostate cancer response to radiotherapy—before and after	*In vivo*	Multiple values for different features reported; approx. 70–114, generally around 80–90					Median age 66; min: 54; max: 77
(36)	Prostate	Prostate cancer detection and differentiation	*In vivo*	79 ± 9 (cancer), 124 ± 38 (peripheral zone), 87 ± 7 (central gland)					63 ± 6 (42‐73)
Skin									
(37)	Epithelium—skin	Identification and characterization of skin layers	*In vivo*/phantoms					Stratum corneum: = 135 ± 10, epidermis = 347 ± 27, papillary dermis = 356 ± 22, reticular dermis = 290 ± 10 and other	Seven normal subjects, five women, two men with mean SD of 32 ± 6 years
Kidney									
(38)	Adrenal gland	Differentiation between pheochromocytoma and other malignant and benign changes in an adrenal gland	*In vivo*	Only stated as relative to cerebrospinal fluid intensity (CSF) and abdominal organs					74, 39 women, 35 men
Liver									
(44)	Liver	Classification of benign and malignant liver lesions, including carcinomas	*In vivo*	Benign: hemangioma: 124.3Cyst: 1007FNH: 62.8Abscess: 406.8Cystadenoma: 459Malignant: metastasis: 65.3 **HCC (carcinoma): 59.1**Hemangioendothelioma: 64.9Cholangiocarcinoma: 55.7Cystadenocarcinoma: 117.5					73 (34 men, 39 women)/54.2 (18-84)
(40)	Liver	Differentiation between different types of lesions with T2 and T1/T2 ratio	*In vivo*, phantoms	Cysts: 371 ± 118Hemangiomas: 204 ± 70 **HCCs/metastases: 83 ± 17**				Cysts: 691 ± 215Hemangiomas: 653 ± 152 **HCCs/metastases: 609 ± 133**	36 (22 men, 14 women)/60 (30-86)
(41)	Liver	Differentiation of liver lesions with T2	*In vivo*	Liver: 54 ± 8FNH: 66 ± 7Malignant lesions: 85 ± 17Hemangiomas: 155 ± 35Cysts: 583 ± 369					52 (28M, 24F) with 114 lesions
(42)	Liver	Differentiation of malignant and benign liver lesions	*In vivo*	Malignant: 40–102, mean 73Hemangiomas: 68–233, mean 165					34 (26M, 8F)/55 (38-70)
(43)	Liver	Differentiation between HCCs and radiation effects	*In vivo*	Liver parenchyma: 42 ± 1.60Irradiated area: 56.4 ± 3.19 **Hepatocellular carcinoma: 58.7 ± 3.19**					X/65
(44)	Liver	Differentiation of lesions with combined quantitative and qualitative analysis	*In vivo*	Liver: 53 (41-74)Solid tumors: 84.1 (54-148)Other lesions: 250.5 (82-1241) **HCC: 75.3 (56-91)**					292 lesions in 168 patients (80M, 88F)/53 (17-83)

### 4.3 Information required in the review

The content required by review rules was generally provided, which means reporting the mean or median T_1_/T_2_ value with standard deviation. A single exception was a study reporting only relative signal intensity between adrenal lesions and abdominal organs (38), but it provided interesting insights anyway. Most of the studies utilized magnetic fields of strength of 1.5 or 3 T. Other field strengths were rather uncommon—single cases of 2 T (37) and 4.7 T (52). The MRI scanner model was always reported, in most cases together with coil type and software used for later data analysis.

### 4.4 Additional information

Age—at least mean or median—of participants was always stated, as well as sex (for mixed groups). Only in a single study (31), the authors tried to classify analyzed tissues and reported classification parameters. Otherwise, when investigating differences between tissues [all except (37)], a test of significance was performed, with p-values reported afterward.

The publications chosen for the detailed review are listed in an attachment (*ReviewResults.ods)*.

Due to anatomical and physio-chemical differences between human tissues, all original publications included in the review were grouped by tissue type or organ type. Because of special interest in them, the breast and selected other tissues were summarized separately from other tissues. Because of special conditions present in cell cultures and their lack of resemblance to the real tissue environment, any studies using cell cultures had to be discarded, even when they used breast cancer cells (52).

### 4.5 Methodology

Multiple factors can affect the results of relaxation time analysis. Some of them result from equipment choice and experimental conditions, such as strength of magnetic field used or type of coil. There are also other nuances present, important from a diagnostic point of view.

Two scenarios were the most common among analyzed studies: differentiating between tissue types (normal/pathological or organ parts, e.g., white and gray matter) or comparing results before and after therapy. To do so, different approaches were applied, some of them strongly susceptible to human error. As seen in **Table 2**, often actions were taken to eliminate risk of a radiologist mistakenly selecting an incorrect region for analysis. In most cases, another type of MRI image or modality (CT) was used to identify the region of interest, which then underwent analysis and could potentially be refined based on the results.

Only sporadically, authors mentioned the stage of the menstrual cycle in the case of breast studies, which might affect T_1_ or T_2_ values obtained due to periodic variations in tissue structure in this area. Such processes might affect not only normal experiments but also those using contrasting agents (53) (**Table 5**).

**Table 5:** Relaxation times reported for epithelial tissue.

	Relaxation time [ms]							
	Breast							Lung
	Identification (depending on the type of pathological changes)		Invasive ductal and lobular carcinomas				Normal	Pathological (pneumonia)
			NAC responders		NAC non-responders			
	Benign	Malignant	Pathological before treatment	Pathological after treatment	Pathological before treatment	Pathological after treatment		
T2	Approx. 84-103	Approx. 77-82	80.93 ± 14.4	63.18 ± 8.37	84.57 ± 6.06	74.62 ± 2.32	41 (28-49) (median)	66.5/74.3/79.5 (median)

#### 4.5.1 Tissues other than epithelium

A basic review (consisting of abstract, methodology, and conclusion scan) of relevant literature showed a variety of applications of relaxation time analysis. The most commonly studied subjects were the nervous system, especially the brain, and connective tissues such as cartilage (**Table 6**).

**Table 6:** T2 values reported by prostate studies.

		Relaxation time [ms]			
		Study A—1.5 T, exterior torso coil		Study B—3 T, endorectal coil	Study C—3T, cardiac coil
		Before therapy	Week 8	Identification of regions	Identification of regions
T2	Prostate	86 ± 10	78 ± 4	–	–
	Central gland	78 ± 8	76 ± 5	–	87 ± 7
	Peripheral zone	114 ± 27	89 ± 13	149 ± 49 (82-290)	124 ± 38
	Tumor	82 ± 15	75 ± 9	97 ± 23 (62-177)	79 ± 9
	Transition zone	–	–	125 ± 26 (84-186)	–

Techniques used for quantitative imaging differed strongly depending on the body area. Some studies implemented relatively simple protocols, using general-purpose whole-body coils (54), whereas others used complex and tailored approaches to cardiac (55–57) or vessel imaging (58). Moderate magnetic fields dominate the literature, such as 1.5 or 3 T, but attempts were made to visualize human tissue at ultra-low or ultra-high fields, e.g., imaging of the hippocampus at 7 T (59) or of the meniscus at 9.4 T (60). Similarly, in the case of gray matter (61) or prostate study mentioned further (33), this time using very low fields, field manipulation is used to achieve better contrast between features of interest, which might be more visible in specific conditions.

A multiplicity of qMRI applications exists, ranging from the analysis of wear and tear of cartilage with age (60) to assessment of pathological changes responsible for conditions such as Alzheimer’s or Parkinson’s diseases (62). Relaxation time analysis is often applied to visualize abnormalities present in the brain, and instead of being used as a direct measure of tissue state (healthy/abnormal), imaging is used to calculate organ part volumes, which in neurology or cardiology are considered important indicators of health. Sometimes different sequences showed different efficiencies depending on the organ part imaged, as in the case of one brain study (63).

Not only T1 or T2 analysis was often performed, but correlations were searched for between them and other parameters, such as ADC (64); T2*, T1rho, T1, and T2 obtained after contrast medium application (non-native relaxation times), diffusion tensor or magnetization transfer imaging (65); or optical coherence tomography results (66). Such measurements complemented the diagnosis made by histological or macroscopic sample assessment.

#### 4.5.2 Epithelium, especially in the breast

Out of 59 accepted original publications, 18 were related to epithelial tissue, including two describing the breast. Apart from those, one publication described breast adipose tissue properties. Because of only these few positions, a decision was made to include other tissues in the comparison, such as the prostate and lung. Although lung parenchyma does not seem to be very similar to breast epithelial regions, there might exist scientific background suggesting genetic likeness between them (67–69).

Studies of epithelial lung and breast tissue did not seem comparable. The same magnetic fields were used, but experimental setups and results differed significantly. As seen in **Table 7**, T_2_ ranges of epithelium do not overlap for breast and lung. On the other hand, means of T_2_ registered for breast epithelium show significant differences (according to corresponding publications), but actual results still overlap, even when only standard deviations are accounted for. That makes any clustering attempts difficult.

**Table 7:** Review inclusion and exclusion criteria for original studies.

Inclusion criteria	qMRI
	Human tissues *in vivo*/*ex vivo*
	T_1_ and/or T_2_ time calculation
	Full text available
	Original publications since the year 2000
	Final publication stage
	Languages: English
Exclusion criteria	Animal tissues or phantoms, cell cultures
	Use of contrasting agents
	No free or academic access
	MRS, NMR, CT, etc.
	Only parameters other than T_1_/T_2_ (T_2_*, T_1_rho, etc.)
	Only T_1_/T_2_-weighted images
	Case reports, case series

The structural and pathogenic likeness is better established between breast and prostate cancer, which makes them often studied together (70–73). Similar treatment approaches may be used for both (74).

All studies regarding prostate reported the significance of differences between T2 values of at least some analyzed areas, as shown in **Table 4**. There was no straight correlation between magnetic field strength and measured relaxation times, but their values suggest more similarity to breast tissue than the lung epithelium showed.

There was no consensus on experimental design in this group of studies, but instead, different types of coils were used depending on external circumstances or other choices.

One novel (for breast) publication was found (75), regarding the calculation of T1/T2 ratios in breast tissues. Unfortunately, it seems that a contrasting agent was used during the scan. It suggests that T1/T2 ratios might change proportionally and be significantly correlated with pathological breast cancer stage. The majority of studies, reports, and analyses were based only on one of two constants, which is shown in **Table 7**. A similar approach, based on T1/T2 ratios, was earlier used in 2006 regarding liver lesions (40), and this one was included in the detailed review as the authors did not use contrasting agents.

The study regarding breast adipose tissue was included in this part because, while it might not be an area susceptible to typical breast carcinomas, it might have the potential to affect relaxation measurements due to its abundant presence. Additionally, there was a chance that a significant difference in relaxation times between epithelium and fat would occur, which could help during ROI selection and tissue differentiation. A series of T1 and T2 values was reported for temperatures ranging from 25°C to 65°C with approximate T2 values for body temperature (37°C) between 65 and 70 ms. The T1 range was between 270 and 320 ms for 37°C. Unfortunately, there were no T1 values reported for breast to compare them with.

In addition to prostate and lung epithelium, other organs were included in this part if their corresponding carcinomas were studied, as they originate from epithelial tissue. Experiments regarding healthy kidney or liver tissue were considered not related to the main topic.

Publication by Cieszanowski et al. (41) provides an exhaustive insight into techniques used for quantitative imaging of liver lesions.

One study (42) presented a slightly different approach than others because not only T2 values were compared but also changes in signal intensity along the entire timeline. Intensity at certain echo times showed better clustering potential than others, as well as relative intensity change compared with the initial tissue state.

## 5 Conclusions

Reporting only one of the constants might be caused by limited resources or assessment of other parameters than the most known ones. There might be some beliefs present, suggesting better efficiency of one or another parameter when differentiating tissues, as considering the number of available publications, it cannot be at this point verified whether any of them is better than the other. In such a situation, it could be profitable to search for correlations between different tissue behaviors instead of sticking to one. It is also possible that many teams tried to analyze both T1 and T2 data, but negative results could have lower chances of getting published than positive findings, as they do not seem as important or revolutionary. Disappointing or inconclusive results were rarely reported (5 out of 59), suggesting areas in which relaxation analysis is, at least currently, not efficient (58, 76–78). Otherwise, multiple reports of high clustering efficiency have been reported.

In cases when the menstrual cycle was not considered or otherwise noted, it could be a source of high intrapatient variation in breast cancer and healthy tissue studies.

Only two studies reported the use of T1/T2 ratios, one examining the liver (without contrasting agents) and the other breast (with contrast), but presented classification results suggest that such an approach might be worth investigating. It is not common nowadays, especially since most researchers perform only one kind of analysis; it is T1 or T2 only, sometimes supported by other sequences or modalities (ADC, CT, T1W, T2W). Classification sensitivity and specificity were much higher for the T1/T2 ratio (close to 100%) than for any of these parameters used separately (~80%).

According to an analyzed study of liver lesions (39), relaxation time analysis has high potential in differentiating between benign and malignant lesions, for example telling apart carcinomas from cysts and hemangiomas. For a more detailed analysis, especially of solid tumors, additional means are necessary, such as dynamic studies with contrasting agents or confirmation by biopsy. qMRI could be thus used just as one of the steps on the way to presurgical diagnosis and treatment choice.

While there was only a single study that mentioned how a patient’s activity before MR scan affects obtained T2 values (50), it should be noted that preexamination procedures might affect studies of different organs depending on mechanical load, oxygen exchange, or blood flow. Additionally, the treatment of *ex vivo* samples could also leave its mark on the samples studied. Such samples also tend to be studied at lower temperatures than body temperature, so correction for that factor could be necessary when attempting comparisons with *in vivo* studies.

Based on analyzed studies, the choice of protocols, technologies, and tissue parameters to assess should be made based on previous publications, which might suggest which approaches have proved successful and what did not work. There are no universal rules for qMRI as too many factors need to be considered when designing an experiment, and thus authors need to try and make informed choices regarding qMRI of any tissue or organ. Some “good practices” are recognized in certain applications, especially for brain and cardiac imaging, so they should be researched beforehand.

## Data availability statement

The raw data supporting the conclusions of this article will be made available by the authors, without undue reservation.

## Author contributions

Conceptualization, MaM; DA; JS, DB-A; MiM; methodology, MaM; DA; JS, D-BA; MiM; validation, MaM; DA; JS, D-BA; MiM; formal analysis, MaM; DA; JS, D-BA; MiM; resources, MaM; DA; JS, D-BA; MiM; writing-original draft preparation, MaM; DA; JS, D-BA; MiM; writing—review and editing, MaM; DA; JS, D-BA; MiM; supervision, MaM; DA; JS, D-BA; MiM. All authors contributed to the article and approved the submitted version.

## Funding

The study was co-financed by the European Union from the European Regional Development Fund under RPO (Regional Operational Programme) for Podkarpackie Region for 2014-2020 “Competitive and Innovative Economy” RPPK.01.02.00-18-0012/18-00 “R+D works on developing expert system supporting data analysis obtained from ﻿breast cancer tissue using MRI”.

## Conflict of interest

Authors MaM, JS and MiM were employed by SoftSystem Sp. z o.o.

The remaining authors declare that the research was conducted in the absence of any commercial or financial relationships that could be construed as a potential conflict of interest.

## Publisher’s note

All claims expressed in this article are solely those of the authors and do not necessarily represent those of their affiliated organizations, or those of the publisher, the editors and the reviewers. Any product that may be evaluated in this article, or claim that may be made by its manufacturer, is not guaranteed or endorsed by the publisher.

## Abbreviations

BOLD MRI, blood oxygenation level-dependent MRI; CT, computer tomography; DCE-MRI, dynamic contrast-enhanced MRI; DWI, diffusion-weighted imaging; FSE, fast spin echo; MRI, magnetic resonance imaging; MT, magnetization transfer; PAI, photoacoustic imaging; PET, positron emission tomography; qMRI, quantitative magnetic resonance imaging; ROI, a region of interest; T_1_w, (images) T_1_-weighted; T_2_w, (images) T_2_-weighted.
